# Complement component C5a induces aberrant epigenetic modifications in renal tubular epithelial cells accelerating senescence by Wnt4/βcatenin signaling after ischemia/reperfusion injury

**DOI:** 10.18632/aging.102059

**Published:** 2019-07-08

**Authors:** Giuseppe Castellano, Rossana Franzin, Fabio Sallustio, Alessandra Stasi, Barbara Banelli, Massimo Romani, Giuseppe De Palma, Giuseppe Lucarelli, Chiara Divella, Michele Battaglia, Antonio Crovace, Francesco Staffieri, Giuseppe Grandaliano, Giovanni Stallone, Pasquale Ditonno, Paolo Cravedi, Vincenzo Cantaluppi, Loreto Gesualdo

**Affiliations:** 1 Nephrology, Dialysis and Transplantation Unit, University of Bari “Aldo Moro”, Bari, Italy; 2 Department of Basic Medical Sciences, Neuroscience and Sense Organs, University of Bari “Aldo Moro”, Bari, Italy; 3 IRCCS Ospedale Policlinico San Martino, Genova, Italy; 4 Urology, Andrology and Renal Transplantation Unit, University of Bari, Bari, Italy; 5 Veterinary Surgery Unit, Department of Emergency and Organ Transplantation, University of Bari, Bari, Italy; 6 Nephrology, Dialysis and Transplantation Unit, Department of Medical and Surgical Sciences, University of Foggia, Foggia, Italy; 7 Department of Medicine, Translational Transplant Research Center, Icahn School of Medicine at Mount Sinai, New York, NY 10029, USA; 8 Nephrology and Kidney Transplantation Unit, Department of Translational Medicine, University of Eastern Piedmont “A. Avogadro” (UPO), Novara, Italy

**Keywords:** acute kidney injury, DNA methylation, complement cascade, renal Inflammaging

## Abstract

Epigenetic mechanisms, such as DNA methylation, affect tubular maladaptive response after Acute Kidney Injury (AKI) and accelerate renal aging. Upon ischemia/reperfusion (I/R) injury, Complement activation leads to C5a release that mediates damage; however, little is known about the effect of C5a-C5a Receptor (C5aR) interaction in Renal Tubular Epithelial Cells (RTEC).

Through a whole-genome DNA methylation analysis in cultured RTEC, we found that C5a induced aberrant methylation, particularly in regions involved in cell cycle control, DNA damage and Wnt signaling. The most represented genes were *BCL9*, *CYP1B1* and *CDK6*. C5a stimulation of RTEC led to up-regulation of SA-β Gal and cell cycle arrest markers such as p53 and p21. C5a increased also *IL-6*, *MCP-1* and *CTGF* gene expression, consistent with SASP development. In accordance, in a swine model of renal I/R injury, we found the increased expression of Wnt4 and βcatenin correlating with SA-β Gal, p21, p16 and IL-6 positivity. Administration of Complement Inhibitor (C1-Inh), antagonized SASP by reducing SA-β Gal, p21, p16, IL-6 and abrogating Wnt4/βcatenin activation.

Thus, C5a affects the DNA methylation of genes involved in tubular senescence. Targeting epigenetic programs and Complement may offer novels strategies to protect tubular cells from accelerated aging and to counteract progression to Chronic Kidney Disease

## INTRODUCTION

Acute Kidney Injury (AKI) is a frequent condition in hospitalized patients and is mainly caused by Ischemia/Reperfusion (I/R) injury, sepsis or nephrotoxic drugs [1]. Currently, AKI is associated with unacceptably high mortality [1] or development of Chronic Kidney Disease (CKD); therefore, there is an urgent need to develop new therapeutic strategies capable to interfere with detrimental effects of AKI and inhibit AKI-to CKD transition [2, 3].

Recent studies have suggested a new hypothesis on pathogenesis of I/R induced-AKI based on epigenetic modification such as DNA methylation and histone acetylation [4–6]. The genome interacts with several environmental factors such as nutrients, pathogens, drugs and toxins that can modify the chromatin condensation to make specific genes accessible or not accessible to transcription factors, thereby extensively regulating gene expression. These modifications become stable and heritable upon mitosis and have been associated to the risk of AKI, allograft rejection after transplantation [7] or transition to CKD [8–10].

DNA methylation is catalyzed by enzyme called DNMT (DNA methyl transferase) and occurs in region highly rich in dinucleotide Cytosine-Guanine called CpG islands, localized above all in the promoters but also in intra and inter-genic regions. High methylation of promoter is associated to a closed chromatin, then to transcriptional silencing [11]. Recently, DNA methylation modifications have been shown also to accelerate renal aging [12]; interestingly, hypermethylation of Klotho promoter, the principal anti-aging and renoprotective factor, led to a reduced Klotho gene expression with a significant association with CKD severity [10].

To date, epigenomic modifications offered drug targets already clinically available in the treatment of different diseases such as cancer; moreover, they showed efficacy also in experimental model of renal fibrosis [13, 14]. Recently, a whole kidney methylome was characterized in a mouse model of I/R describing the central role of DNA regulatory enzyme (DNMTs and Tet methylcytosine dioxygenase) in promoting renal fibrosis, inflammation and apoptosis [15]. Nevertheless, untargeted DNA demethylation therapy (with DNMTs-inhibitors) has not been successful in the attenuation of I/R induced fibrosis, highlighting the need of highly specific target genes [16].

In the pathophysiology of I/R injury, Complement system plays a pivotal role and the C1 esterase inhibitor C1-INH (C1-Inhibitor) is a promising strategy to prevent I/R damage in kidney transplantation and other forms of AKI [17, 18]. During I/R injury, Complement activation after C3 convertase generation led to the release of pro-inflammatory anaphylatoxin C5a that binds to C5a receptors expressed on monocytes, neutrophils and renal resident cells [19–21]. In the present study, we aimed to investigate the effect of C5a on human renal tubular epithelial cells (RTEC) by a genome-wide DNA methylation analysis.

## RESULTS

### C5a induces DNA methylation changes in RTECs

**Figure 1:**
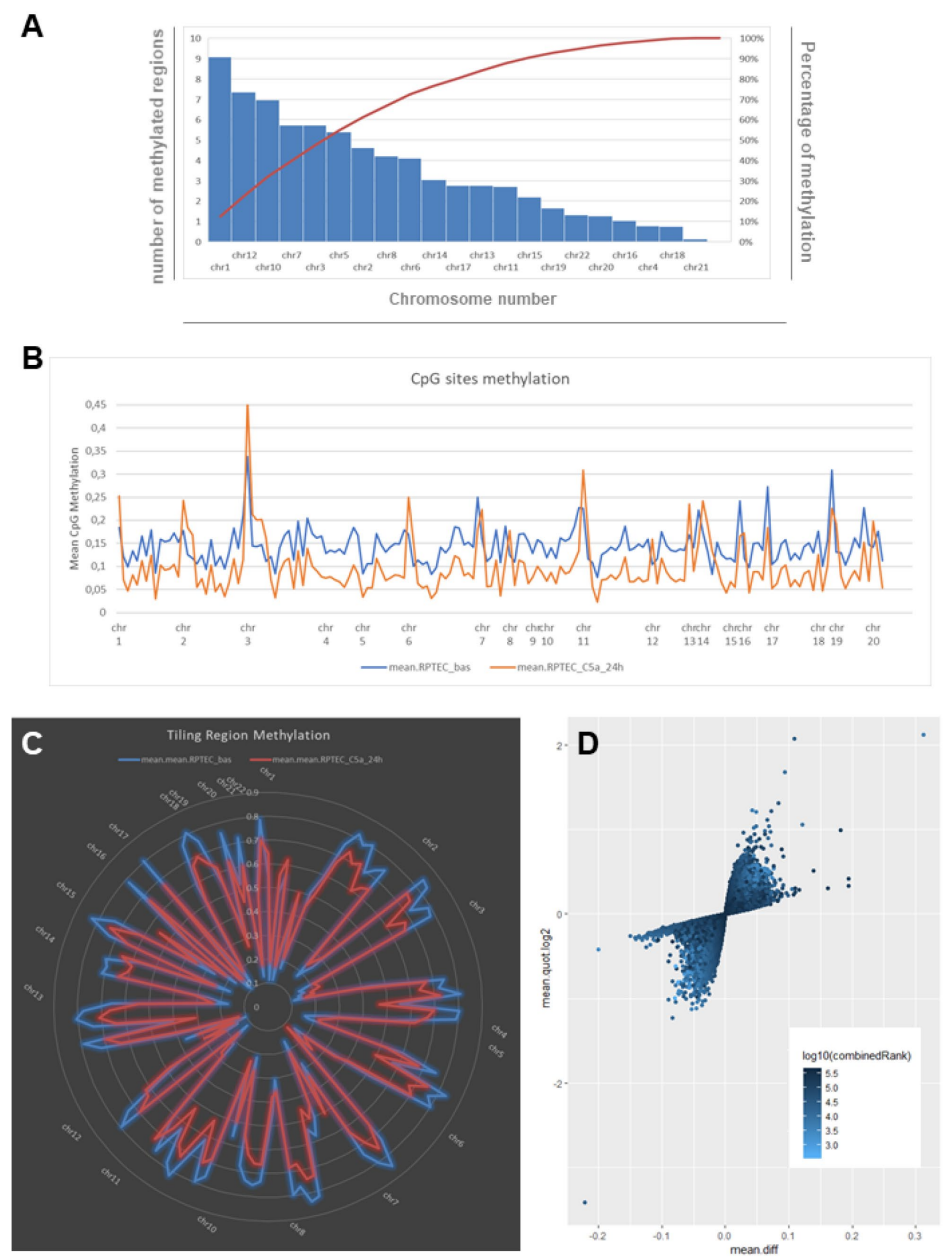
**C5a-associated changes in DNA methylation as indicated by whole-genome bisulfite assay in RTEC.** (**A**) Chart showing the number and the frequency of methylated regions (tiling regions) identified in RTEC at basal level. The left vertical axis represents the number of methylated regions per each chromosome. The right vertical axis indicates the cumulative percentage of the total number of occurrences. The red concave curve is the cumulative function indicating that the 50% of the total methylated regions in RTEC are covered by chromosomes 1, 12, 10, 7 and 3. (**B**) DNA methylation levels at the single CpG sites for RTEC at basal level (blue line) and for RTEC stimulated by C5a (orange line). C5a decreased the DNA methylation in the overall genome. (**C**) Graph showing the mean DNA methylation levels of tiling regions, shared for chromosomes, in RTEC (blue line) and in C5a-stimulated RTEC (red line). The central axis shows the mean β methylation value. (**D**) Scatterplot of the CpG site methylation comparison, colored according to the combined ranks of a given site. Values are represented as mean differences (mean.diff) between stimulated and unstimulated RTEC for each CpG site. (Combined rank: difference in mean methylation levels of stimulated and non-stimulated RTEC, the quotient in mean methylation and the t test are ranked for all regions. This value aggregates them using the maximum, i.e. worst rank of a site among the three measures.)

To study whether Complement could modulate DNA methylation in renal cells, we stimulated human RTECs with C5a anaphylatoxin for 24 hours and performed a whole-genome DNA methylation study. Methylation analysis was performed for single CpGs and also for sets of predefined genomic regions such as CpG islands, promoters and tiling regions (5-kb spanning regions). In untreated cells (indicated as basal), the highest number of methylated tiling regions were found in chromosome 1, 12, 10, 7 and 3. They cover the 50% of the total tiling regions, whereas chromosomes 4, 18 and 21 contained the fewest methylated regions (Figure 1A). C5a stimulation decreased the DNA methylation in the overall genome when we consider the single CpG sites (Figure 1B). In particular, considering the tiling regions, most DNA regions were hypomethylated and located in specific chromosomes (Figure 1C, Supplementary Table 1).

We then performed differential methylation analysis according to the combined Rank parameter and found 74 hypermethylated and 320 hypomethylated CpG sites in RTEC stimulated with C5a (Figure 1D). Next, after the differential methylation analysis of the tiling regions we selected the best 150 combined ranks having a threshold of Δβ>0.05. We found 142 regions, 88 hypomethylated and 54 hypermethylated (Supplementary Table 2). In addition, when we analyzed differentially methylated CpG island, we found only 3 island hypermethylated and 8 island hypomethylated by C5a (Supplementary Figure 4F).

In order to gain insight into the biological mechanisms involved in the response to C5a, we performed a pathway and a gene ontology (GO) analysis of genes spanned in the identified DNA regions (Supplementary Figure 1). The pathway analysis of regions hypo- and hypermethylated by C5a showed the involvement of biological processes as “Aryl Hydrocarbon Receptor Signaling”, “cell cycle: G2/M DNA damage checkpoint regulation” and “Wnt/βcatenin pathway” (p=0.019, 0.027 and p=0.033, respectively). In particular, we found three hypomethylated genes as central node of the networks: *BCL9, CYP1B1* and *CDK6* (Supplementary Figure 2). These genes are involved in the cell cycle progression and apoptosis. In addition, also the GO analysis showed an enrichment of DNA regions containing genes involved in the cell cycle checkpoint (Supplementary Figure 3).

**Figure 2:**
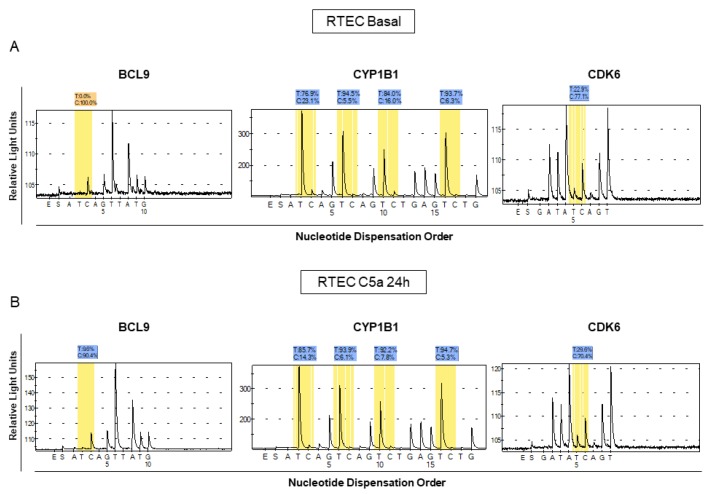
**Analysis of methylation levels for BCL9, CYP1B1 and CDK6 in different lots of C5a-stimulated RTEC compared to the respective basal conditions.** Pyrosequencing assays were performed on the same regions that we found to be methylated in the whole-genome assay. The pyrosequencing assays confirmed that the DNA of these regions were differentially methylated in C5a-stimulated RTEC (**B** panel) compared to basal condition (**A** panel). Representative pyrograms show methylation levels of the indicated CpG sites in the gene promoter.

We then validated the methylation status of the three genes by pyrosequencing (Figure 2A, 2B). We confirmed that *BCL9*, *CYP1B1* and *CDK6* genes were hypomethylated by C5a (p<0.05) with mean differences in methylation levels of about 8-10% (Figure 2A, 2B). In particular, the *CYP1B1* region contained multiple CpG sites most of which were concordantly hypomethylated.

Overall, these data demonstrated the functional effect of the C5a anaphylatoxin to re-program in-depth the tubular cells epigenetic state, promoting a global hypomethylation in different chromosomes. In addition, the DNA methylation changes were consistent with cell cycle pathway.

### C5a induced DNA methylation regulating *BCL9, CYP1B1* and *CDK6* gene expression

**Figure 3:**
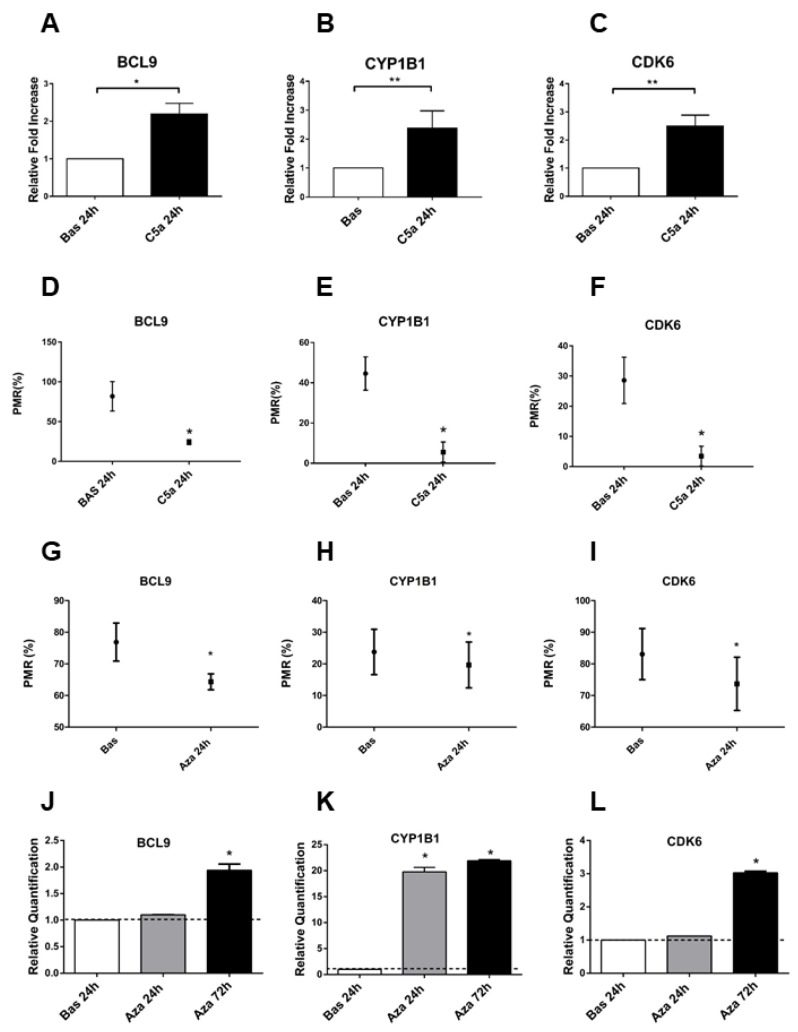
**BCL9, CYP1B1 and CDK6 gene expression is regulated by the DNA methylation.** (**A**–**C**) Gene expression of BCL9, CYP1B1 and CDK6 evaluated by qRT-PCR in the C5a stimulated-RTEC and compared to normal RTEC cultured for 24h. The gene expression validated the genes analyzed for the methylation using the pyrosequencing assay. Expression levels were significantly different in C5a-stimulated RTEC compared with normal cells. Gene expression levels were normalized to the housekeeping gene GAPDH. (**D**–**F**) BCL9, CYP1B1 and CDK6 methylation levels of C5a-stimulated RTEC compared to basal condition. Results are means±SD, n=3. (**G**–**I**) Gene expression of BCL9, CYP1B1 and CDK6 of C5a stimulated-RTEC not treated or treated with 1 μM 5-aza-2’-deoxycytidine. The DNA demethylation agent reduced the methylation levels in the promoters of the three genes. The DNA methylation status of these three DNA regions was determined by qMSP real-time analysis. The degree of fully methylated molecules at a specific locus was expressed as a PMR index. The percentage PMR was calculated as described in the Materials and methods. Qiagen methylation control DNA was used as full methylated reference. (**J**–**L**) Gene expression of BCL9, CYP1B1 and CDK6 of C5a stimulated-RTEC not treated or treated with 1 μM aza. Results are means±SD, n = 3.*p<0.05.

To confirm the critical role of DNA methylation in regulating the *BCL9*, *CYP1B1* and *CDK6*, we assessed the gene expression from C5a-stimulated RTEC compared with untreated cells (Figure 3). The genes were differentially expressed in C5a-stimulated RTEC; their expression was inversely associated with the relative DNA methylation (Figure 3D–3F): *BCL9*, *CYP1B*1 and *CDK6* genes were hypomethylated with a significant up-regulation of their gene expression. (Figure 3A–3C: mean fold changes of *BLC9* 2.19±0.5, *CYP1B1* 2.38±0.59 and *CDK6* 2.49±0.62, *p<0.05, **p<0.01).

Next, to further validate the regulatory involvement of DNA methylation in *BCL9*, *CYP1B1* and *CDK6* gene expression, we treated RTEC for 24h with the DNA methyltransferase inhibitor 5-aza-2’-deoxycytidine (5-aza, 1μM), a DNA hypomethylating agent. Following 5-aza treatment, we assessed *BCL9*, *CYP1B1* and *CDK6* hypomethylation (Figure 3G, 3H, 3I). Effectively, after 5-aza induced-DNA demethylation, we found that the mRNA expression of *BCL9*, *CYP1B1* and *CDK6* by qPCR significantly increased (Figure 3J, 3K, 3L). These results indicated that gene expression of *BCL9*, *CYP1B1* and *CDK6* is directly regulated by DNA methylation and that C5a is an epigenetic mediator that caused hypomethylation of genes that are involved, at least in part, in the Wnt/βcatenin signaling.

### Characterization of C5a-induced cellular senescence in RTEC

**Figure 4:**
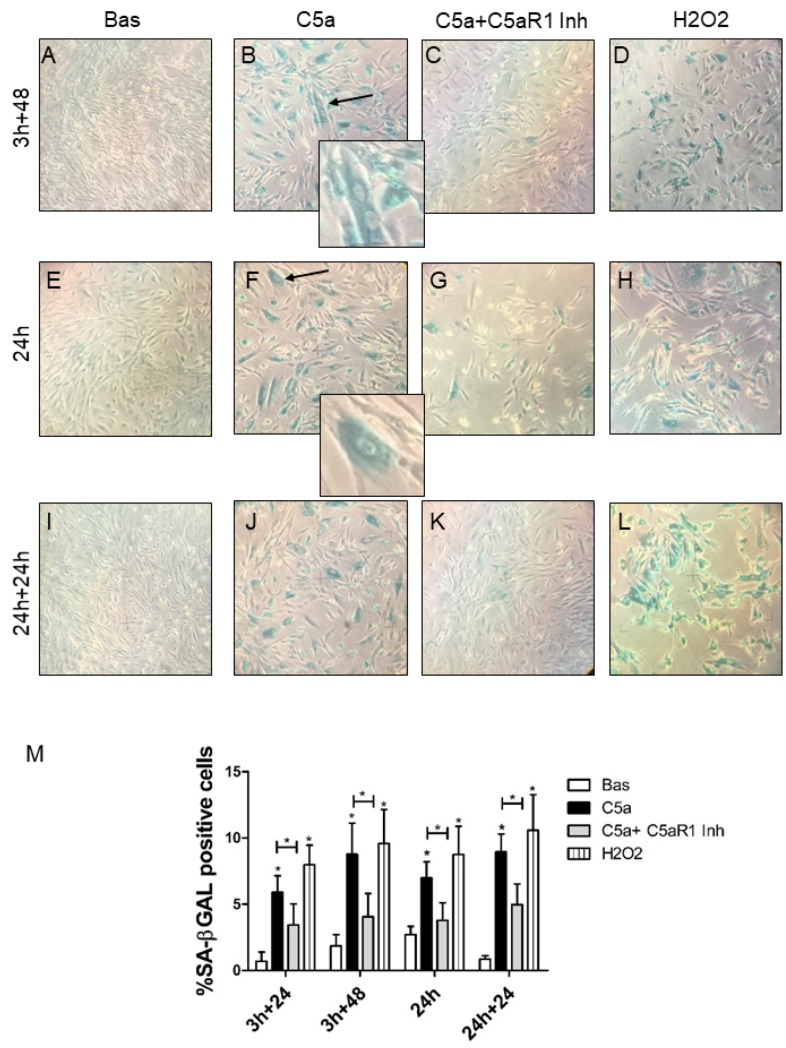
**SA-β Gal staining in RTEC treated with C5a with or without C5aR1 blocking.** SA-β-Gal activity in early passage RTEC exposed to 100 nM C5a for 3h (**B**, **F**) or 24h (**J**). For C5aR1 inhibition, mouse monoclonal anti-C5aR1 was pre-incubated for 1h before the C5a exposure and then maintained in fresh medium for 24h or 48h (**C, G, K**). More SA-β-gal+ cells were observed after C5a exposure, senescent RTEC appeared enlarged and morphologically distinct from the normal cells at the same passage with formation of larger and polynucleated cells (B and F, arrows). Untreated cells are also named as Basal (**A, E, I**). H_2_O_2_ exposed cells were used as positive control of senescence (**D, H, L**). Representative images were acquired by phase contrast microscopy. (**M**) Quantification of SA-β-Gal+ cells cultures. The ratio of cells positive for SA-β-gal activity was calculated by examining five not overlapping fields per condition (6-well plate). The results are presented as the mean ± SD of three independent experiments (*p< 0.05), Magnification 40X.

Because the three validated genes were clustered in Wnt/βcatenin pathway [22, 23] that is involved in aging processes [24], we tested the hypothesis that C5a might directly promote RTEC senescence *in vitro*. We then stimulated RTEC by C5a for 3h and 24h (Figure 4B and 4F, 4J) followed by culture in fresh medium for additional 48h (4B) or 24h (4J); interestingly, C5a stimulated-RTEC acquired a senescent phenotype as determined by significant increase in SA-βGAL positivity (Figure 4B, 4F, 4J). C5a exposed RTEC become larger (Figure 4F, arrow) and polynucleated (Figure 4B, arrow). The inhibition of receptor C5aR1 appeared to significantly protect tubular cells from C5a-induced senescence (Figure 4C, 4G and 4K), indicating that C5a effect was mediated by this receptor. RTEC exposed to H_2_O_2_ (Figure 4D, 4H and 4L) were used as positive control of cellular senescence. Quantification of senescent cells in Figure 4M showed that the induction of C5a mediated RTEC senescence was statistically significant in all the condition tested and also in presence of different time of activation (3h and 24h; *p<0.05, images at 3h+24 not showed).

**Figure 5:**
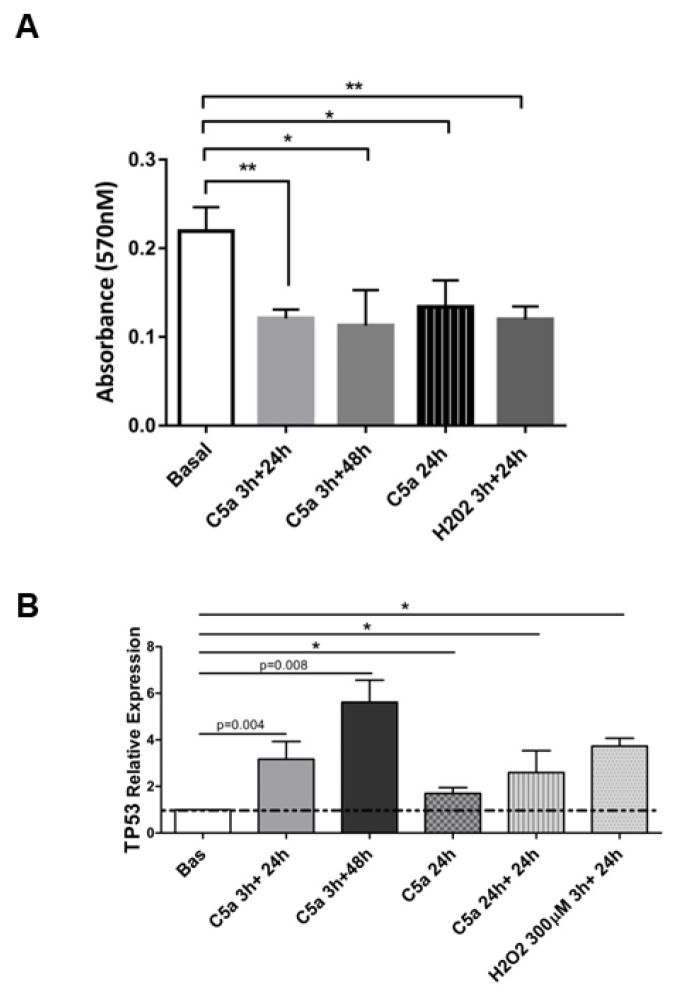
**Altered cell proliferation and p53 expression after C5a exposure in RTEC.** (**A**) MTT assay of RTEC in logarithmic growth phase treated with the same dosage of C5a showed an inhibition of cell proliferation. (**B**) TP53 gene expression level in C5a stimulated RTEC. Data are expressed as the mean ± SD (*p< 0.05 versus basal condition).

Since cellular senescence is characterized by cell cycle arrest, we performed the MTT assay to assess the number of viable RTEC (Figure 5A). C5a led to a reduction in cellular proliferation compared to untreated cells (MTT C5a 24h: 0.134±0.03 vs basal 24h: 0.219±0.02); the inhibition of proliferation was stable even in presence of short C5a stimulation with 48h of culture in fresh medium (C5a 3h+48h: 0.013±0.04 vs basal). RTEC exposed to H_2_O_2_ were used as internal control showing a 54% inhibition of proliferation. In addition, cells counting performed by Trypan blue from the supernatants of cell culture did not indicate significant variations in the viable/dead cells number ratio (data not shown).

Next, we tested the activation of p53 /*TP53* gene pathway that is crucial in regulating premature senescence and cell- cycle arrest [25]; therefore, we performed a cell cycle assay and assessed the expression of *TP53*. Three hours after C5a exposure, we observed a significant increase in p53 transcript that further increased after 24h and 48h of culture (mean fold change: C5a 3h+48h: 5.6±1.64, p=0.008; C5a 3h+24h: 3.17±1.32, p=0.004, versus basal 1±0.0). The p53 expression increase was higher even after a short C5a stimulation (3h) followed by 48h of normal culture in fresh medium (Figure 5B); effectively most of cells were halted in G0/G1 phase of the cell cycle (83% versus 58% of non-stimulated cells), indicating that C5a affected the arrest of the cell cycle (Supplementary Figure 4A).

### C5a induced the development of Secretory Associated Senescence Phenotype (SASP) in RTEC

**Figure 6:**
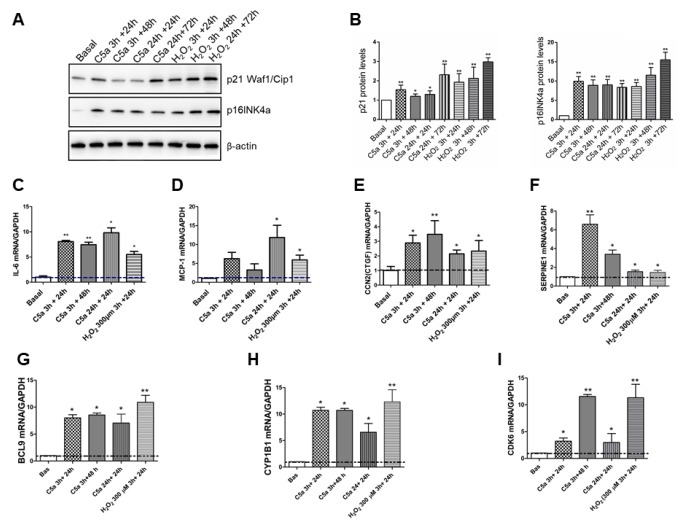
**Cell cycle negative regulators expression levels and SASP pro-inflammatory cytokines transcripts are up regulated by C5a.** (**A**–**B**) Representative p21 Waf1/Cip1 and p16INK4a western blot of C5a-stimulated RTEC and quantification. Protein expression was normalized to βactin. (**C**–**I**) Time course of IL-6, MCP1, CTGF, SERPIN1 (PAI-1), BCL9, CYP1B1 and CDK6 gene expression in RTEC stimulated for 3h or 24h by C5a followed or not by 24h or 48h of normal culture was assessed by qPCR. Data were normalized to GAPDH. *p<0.05, **p<0,01, n=3. H_2_O_2_ (100-300 μm) was used as positive control of senescence.

After p53 activation, cells transiently express p21Cip1/Waf1 [26] a Cyclin-Dependent Kinase Inhibitor (CKI) that can lead to a chronic state of senescence, triggering G1 cell cycle arrest or leading to apoptosis. Therefore, we measured the p21 protein level in C5a stimulated RTEC and we found a significant protein increase after both short time (3h) and longer time (24h) of C5a exposure. (Figure 6A, 6B, p21 C5a 3h+24: 1.45±0.17; p<0.01, C5a 24h+24: 1.28±0.37, p<0.05). In particular, this effect was more evident after 24h of C5a exposure followed by 72h of normal culture. Since p21 could transiently increase after an injury, we assessed also the p16INK4a protein level. Stimulation with C5a significantly induced a constant augment in protein expression of p16INK4a compared to untreated condition (basal) (Figure 6A and 6B).

Subsequently, we investigated the gene expression of IL-6, MCP-1, CTGF and PAI-1, that are chemokines typical associated with the Senescence-Associated Secretory Phenotype (SASP) [27]. As observed for p21 protein, we found a significant increase in *IL-6*, *SERPINE1* (PAI-1), *CCL2* (MCP-1) and *CCN2* (CTGF) after three hours of C5a exposure followed by 24h of normal culture (Figure 6C–6F; mean fold change IL-6: 8.05±0.24; PAI-1: 6.58±1.72 versus baseline, p<0.01). Interestingly, IL-6, PAI-1 and CTGF gene expression levels remained high even after 48h of culture from C5a stimulation. Significant expression increases for all these genes, except MCP1, was found also after 24h of C5a exposure followed by 24h of normal culture. Increase in the gene expression of each pro-inflammatory cytokine was found also by 300 μM H_2_O_2_, that is a stressor inducer of SASP (Figure 6C–6F).

Finally, in order to gain insight in *BCL9, CYP1B1* and *CDK6* genes expression we performed qPCR at different time points. All genes showed a significant up-regulation both after a short C5a stimulation (3h) followed by 24 or 48h of culture in normal medium and after 24h of C5a stimulation followed by 24h of culture. A similar increase was detected after 300 μM H_2_O_2_ exposure (Figure 6G–6I).

Taken together these results demonstrate that C5a can induce tubular senescence *in vitro,* characterized by morphological and functional changes such as growth arrest and acquirement of a pro-inflammatory phenotype named SASP.

### C1-INH modulated Wnt pathway activation during I/R leads counteracting renal senescence

We further investigated whether I/R might promote the development of tubular senescence in vivo. To this aim, we used a swine model of renal I/R injury in which pigs were treated with C1-Inhibitor to modulate Complement activation [28, 29]. We first investigated Wnt4 expression on renal sections obtained at 24 hours after I/R injury since the *in vitro* data showed an aberrant methylation and increased expression of *FZD6* (Supplementary Figure 4B, 4D), a well-known Wnt4 receptor [30].

**Figure 7:**
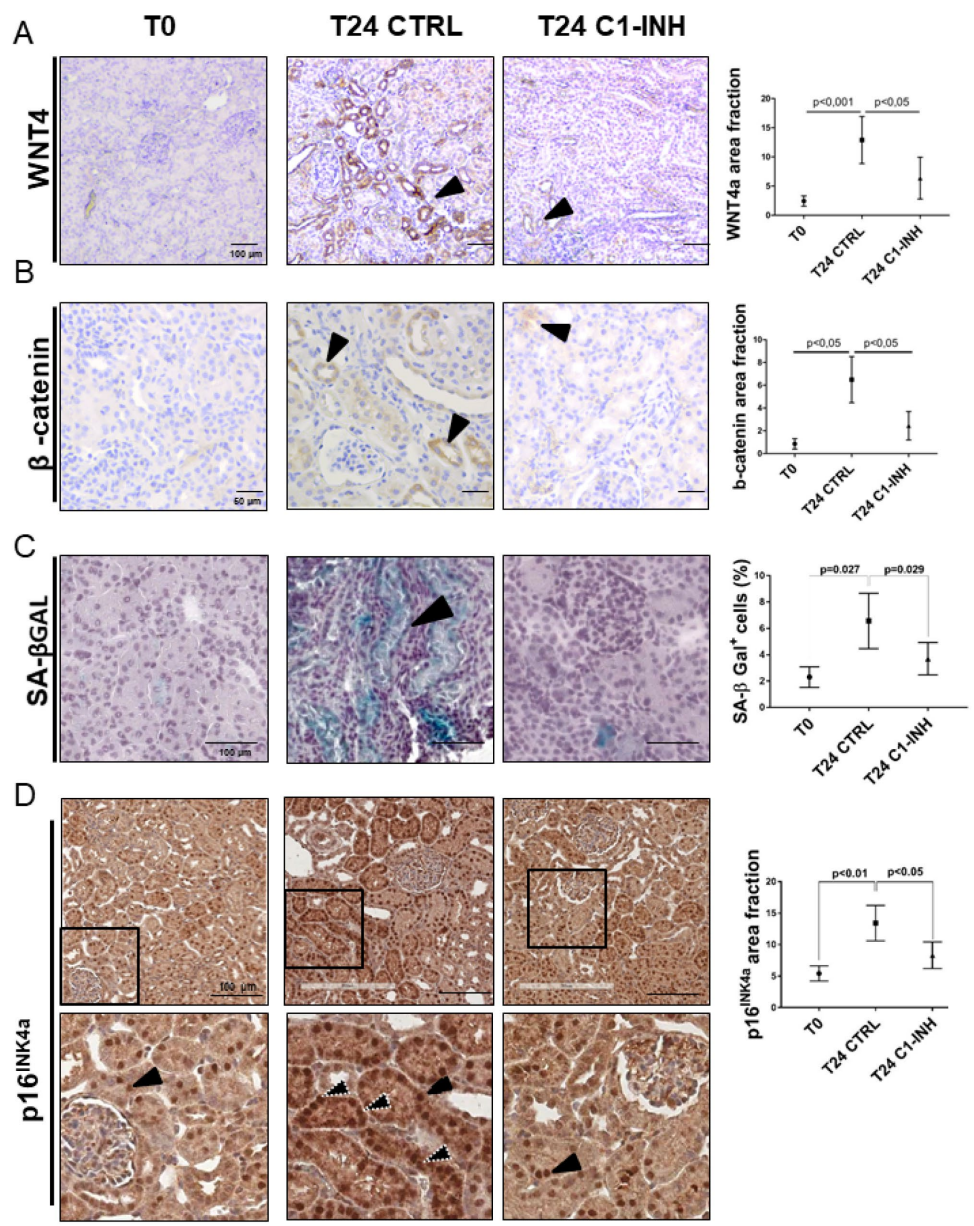
**Wnt4/βcatenin pathway and inflammaging markers are activated in tubular cells after I/R and modulated by C1-INH treatment.** (**A**–**B**) Immunoistochemical stainings showing the tubular Wnt4 and βcatenin increase after 1 day of I/R injury and the C1-INH-mediated modulation. IHC was performed on paraffin kidney sections. Arrows indicate positive tubular staining. (*in the right*) Graphical representation of Wnt4 and βcatenin protein expression level in the different groups. (n=5, p value as indicated, scale bar as indicated).(**C**) Representative SA-β Gal stained kidney tissues revealed an higher number of senescent cells after 24 h of I/R injury compared to T0. Treatment with C1-INH restored SA-β Gal at basal expression. Arrows indicate positive tubular staining on cryo tissues. *(in the right*) Graphical representation %SA-βGal area fraction. (n=5, p value as indicated, scale bar as indicated). (**D**) Representative micrographs indicating p16INK4a protein expression in different groups of swine, as indicated. Boxed areas are enlarged in the bottom of each micrographs. In the T0, p16INK4a had constitutive level and was localized in tubular nuclei (black arrow). Biopsies after 24h from reperfusion (T24 CTRL) showed increased nuclear (black arrow) and cytoplasmic staining (white dotted arrows). C1-INH restored p16INK4a at basal expression; limiting the cytoplasmic p16INK4a expression. (*in the right*) Graphical representation of p16INK4a area fraction in the different groups. (n=5, p value as indicated, scale bar as indicated).

During I/R injury, the protein level of Wnt4 increased compared to T0 (Figure 7A). The expression mainly localized to the apical side (Figure 7A, T24 CTRL arrow) of epithelial tubular cells and was more evident in area of tubular damage characterized by vacuolization and interstitial edema. Moreover, Wnt4 expression was significantly reduced by treatment with C1 inhibitor (Figure 7A, T24 C1-INH). Subsequently, we assessed the Wnt/β-catenin pathway activation by measuring the level of β-catenin activation. One day after I/R injury, an increased number of β-catenin^+^ cells were detected (Figure 7B). Compared with the T0, β- catenin protein was up regulated predominantly in renal tubules of the I/R injured kidney. β-catenin was localized in the cytoplasm and the nuclei of tubular epithelial cells. (Figure 7B, T24 CTRL). After 24h from the reperfusion, relative β-catenin levels were increased compared to the T0, suggesting that induction of Wnt expression would result in an accumulation of β-catenin in injured and senescent kidney. Less β-catenin^+^ cells were present in C1 inhibitor-treated pigs (Figure 7B, T24 C1-INH), again showing the involvement of Complement in the activation of this pathway.

**Figure 8:**
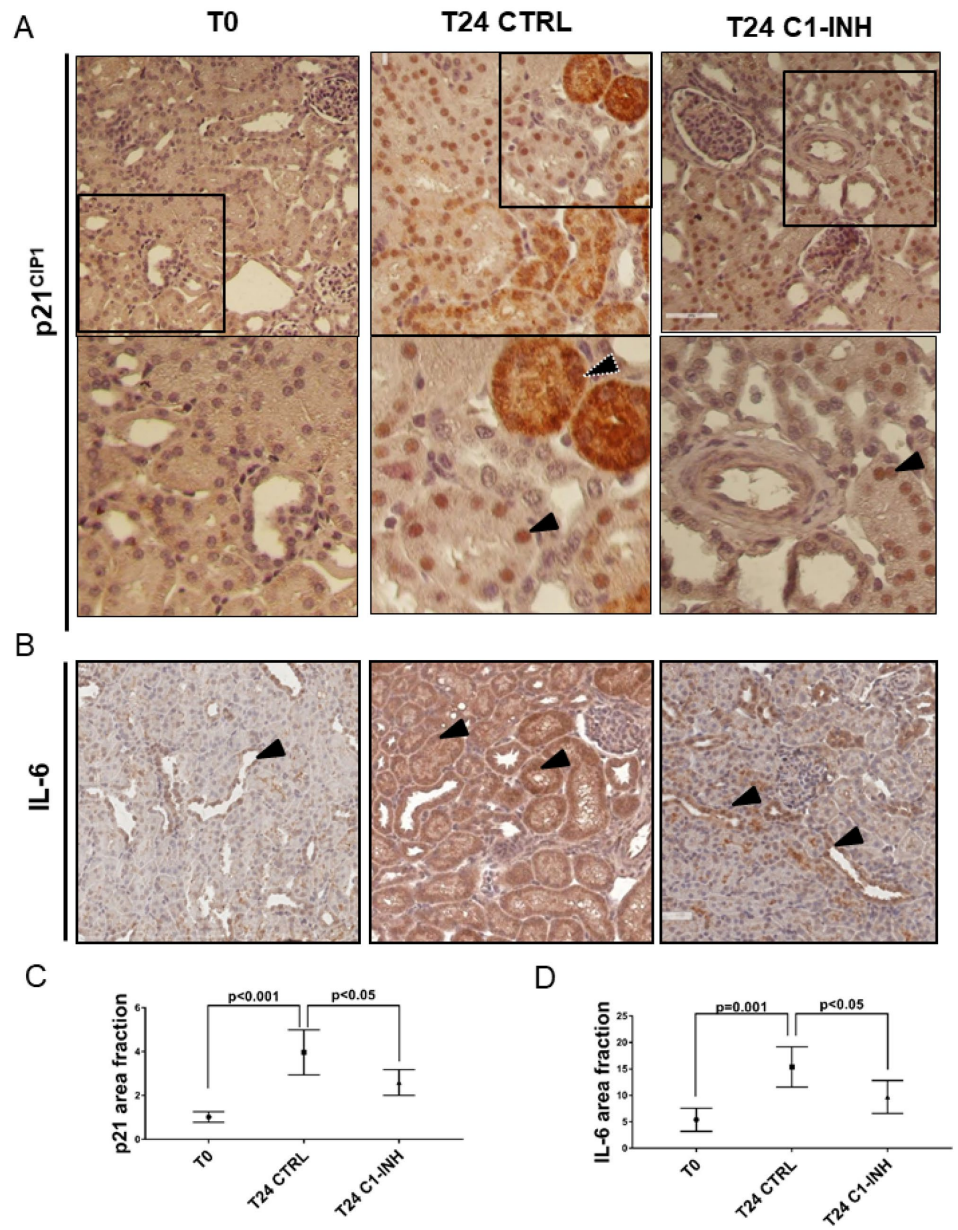
**p21 and IL-6 are induced in tubular cells after I/R and modulated by C1-INH treatment.** (**A**) Representative micrographs indicating p21/CIP1 protein expression in different groups of swine after I/R injury, as indicated. Boxed areas are enlarged at the bottom of each micrographs. Compared to T0, biopsies after 24h from reperfusion (T24 CTRL) showed increased nuclear (black arrow) and cytoplasmic staining (white dotted arrow). C1-INH treatment restored p21 at basal expression; limiting the cytoplasmic p21 expression. (**B**) Representative micrographs show the expression and localization of IL-6, a marker of SASP in T0 (right), T24 CTRL (middle) and T24 C1-INH (right) groups. Arrows indicate positive tubular cells. Compared to T0, biopsies after 24h from reperfusion (T24 CTRL) showed increased IL-6 expression, predominantly at tubular cells. Treatment with C1-INH counteracts IL-6 increase. (**C, D**) Graphical representation of p21 and IL-6 area fraction in the different groups. (n=5, p value as indicated).

To further clarify the final effect of the Wnt activation, we analyzed the tubular senescence by SA β-gal, p16 and p21 staining. As shown in Figures 7C and 7D and 8A (T24 CTRL), the Wnt/β-catenin activation was associated with SA β-gal positivity and increase in tubular p16 and p21 24h after I/R injury, Regards to p16 and p21, we detected beyond the constitutive nuclear staining (black arrow) (Figures 7D, 8A, T24 CTRL], a strong and diffuse cytoplasmic staining (white dotted arrow) that was described to have a negative clinical significance [31–33]. Furthermore, to assess the acquirement of SASP in vivo, we stained for IL-6 (Figure 8B) and found a statistically significant increase, as shown in Figure 8B and 8D.

As shown in Figure 7A and 7B, Complement inhibition significantly abolished the Wnt/β-catenin activation induced by I/R. Consistently with the decreased activation of Wnt/β-catenin pathway by C1-inhibitor, the reduction of Wnt4 was associated to a block of SA-β gal enzymatic tubular activity (Figure 7C), to a reduction of p16 and p21 cytoplasmic expression (Figures 7D, 8A and 8C) and to a downregulation of IL-6 production. (Figure 8B and 8D). These results provide in vivo proof that Complement modulation can reduce tubular senescence by regulating Wnt/β-catenin pathway and inducing SASP in vivo.

## DISCUSSION

In this study, we provide evidences that Complement anaphylatoxin C5a significantly triggers DNA methylation changes in renal tubular epithelial cells. C5a epigenetically induced the expression of genes involved in the Wnt/βcatenin pathway promoting the acquirement of a premature senescence-related phenotype called SASP. We confirmed the occurrence of this pathogenic process in vivo in a swine model of renal I/R injury where the treatment by C1-INH could inhibit the Wnt/βcatenin signaling and the occurrence of tubular Inflammaging.

It is known that Complement is the driving force in the pathophysiology of I/R injury-induced AKI [34, 35] and C5a, the most powerful anaphylatoxin, is able to bind two kind of receptors (C5aR1 and C5aR2), both expressed on renal tubular epithelial cells [36, 37]. Recently, a growing body of evidence suggested that epigenetic mechanisms are involved during acute injury in the transition from AKI to CKD, [13, 38–40]; therefore we reasoned that C5a might have an effect on the DNA methylation profile in tubular cells by regulating the expression of inflammatory or injury factors after I/R. In our study, in order to extensively cover the whole human genome, we analyzed all CpGs regions into promoters, 5’UTR, 3’UTR gene exons and introns. Our observations that C5a induced the hypomethylation of 88 DNA regions and the hypermethylation of 54 DNA sequences are in line with data from literature showing a significant correlation between DNA methylation changes in kidney donors after I/R and the risk to progression to CKD [39]. Interestingly, we found that C5a exposure induced a global genome-wide hypo-methylation (Figure 1B), a DNA pattern that has already been observed in human tissues during aging by several groups [41–44]. This conclusion could be supported by recent evidences showing that Complement has a central role in several aged related disease [45, 46].

Between all the pathways involved in C5a-stimulated RTEC, we showed that Complement changes the methylation level of genes involved in Wnt/βcatenin pathway, the DNA damage response, the cell cycle checkpoint regulation and in particular the Aryl Hydrocarbon Receptor Signaling. Concerning Wnt/βcatenin pathway, [47] it has been demonstrated its significant role in the aging kidney [48, 49] in several experimental models of chronic fibrosis [50–52]. Interestingly, DiRocco Derek P et al, demonstrated that Wnt/βcatenin signaling constitutive activation was sufficient to drive spontaneous myofibroblast differentiation in absence of injury [53], with Wnt4 playing a pivotal role in chronic fibrosis [54].

By network analysis (Supplementary Figure 2), we determined the biological and functional connectivity of all differentially methylated genes and we were able to identify and validate (Figures 2; 3) three main genes as central nodes in the network: BCL9, CDK6 and CYP1B1. These hypomethylated genes functionally belonged to Wnt/βcatenin pathway. BCL9 is an essential co-factor in the Wnt/β-catenin [55]; in addition, the protein kinase CDK6 that regulates the cell cycle is under the control of Wnt [56]. Our findings are in line with previous data that showed firstly a pivotal role of Wnt signaling during AKI [57–60] and secondly a new pathogenic and pro-inflammatory effect of CDK6 during renal I/R injury [61]. Finally, also CYP1B1, a cytochrome P450 monooxygenase involved in estrogen metabolism, can be regulated by β-catenin [62] and can activate Wnt signaling [63–65]. Nevertheless, the CYP1B1 finding, that acts as a well-known oncogenic protein in renal cell carcinoma by the Aryl Hydrocarbon Receptor Signaling suggested an unexplored link between aberrant Complement activation, DNA damage and tumorigenesis [66]. Even if it is known that the C5a/C5aR1 pathway can mediate tumorigenesis in several animal models [66–69], more studies are required to elucidate how Complement activation and C5aR signaling could differentially lead to premature senescence by Wnt pathway or increase susceptibility to cancer after transplantation.

The double presence of Wnt pathway (Supplementary Figure 1) in the most significant biological processes and its pivotal involvement in renal aging [49] led us to investigate the tubular senescence. Cellular senescence is defined as a state of irreversible cell cycle arrest, DNA damage, apoptosis inhibition and a persistent secretion of pro-inflammatory cytokines, which is referred as the Senescence-Associated Secretory Phenotype (SASP) [70]. The growth arrest is maintained by an increase of cell cycle regulators as p16^INK4A^, p21^CIP1^ and p53 [49, 71], the SASP by the cytokines IL-6, MCP-1, CTGF and PAI-1 [72, 73]. Our results demonstrated that Complement is involved in premature senescence in tubular cells and that this event is induced after a short period of stimulation and then became permanent and stable even after the anaphylatoxin removal. This is consistent with results showing the predominant role of C5a in the early phases of I/R injury [74] and with the chronic effect of Complement activation after renal transplantation. In particular, by SA-β-gal assay we found the C5aR1 inhibition was able to attenuate the acquirement of a senescence phenotype, modulating also the C5a-induced enlargement of cell size. These results are in line with several findings showing that C5aR1 inhibition protects kidneys from I/R injury [75, 76].

Recent studies have revealed that Wnt signaling leading to mammalian aging can be induced by Complement component C1q [24] and antagonized by the protein Klotho [77, 78]. Furthermore, Complement severely reduced Klotho level in tubular cells [29]. Thus, we hypothesized that Complement modulation could regulate this vicious cycle that lead to tubular senescence and graft failure. C1-INH is a Complement inhibitor safely used as therapy for hereditary angioedema and antibody-mediated rejection [79] (NCT 01134510, NCT 01035593, NCT 01147302). Our *in vivo* studies confirmed that renal I/R activated Wnt/β-catenin signaling [52, 80] and that accelerates renal aging as showed by p16 and p21 higher level (Figures 7D, 8A T24 CTRL versus T0) [32, 81, 82]. The interesting observation is that C1-INH treatment abrogated Wnt4 and reduced the β-catenin activation. This inhibition led to a reduced tubular senescence as observed by decreased SA-βgal, p16, p21 and IL-6 positivity. These data suggest a consequential correlation between Complement activation, the epigenetically induced Wnt signaling and tubular senescence after I/R injury. Considering the strong link between senescence and fibrosis as long-term consequences of renal graft deterioration, these findings are supported by several studies showing anti-fibrotic role of C1-INH [28, 83, 84].

We recognize that overall our study has some limitations. The methylation data used included only 3 different lots of human RTEC and further investigation is required to increase the number of samples. However, by analyzing one single renal population we obtained results that are not affected by cellular heterogeneity since DNA methylation is cell-type specific. In addition, we evaluated the Wnt pathway modulation in a swine model of renal I/R injury. Even if pigs represents one of the best large animal models for kidney transplantation [85], in a next future human renal biopsies should be investigated.

In conclusion, with the present study we demonstrate for the first time, that C5a can modulate the DNA methylation of specific genes in RTEC and that following Complement exposure, the Wnt/βcatenin pathway was significantly activated, leading to a senescent phenotype. Therefore, understanding of the epigenetic mechanisms underlying the AKI-accelerated renal senescence could pave the way for the use of Complement inhibitors to counteract the progression from AKI to CKD.

## MATERIALS AND METHODS

### RTEC culture and DNA/RNA extraction

RTEC (human Renal Proximal Tubular Epithelial Cells) between 3-5 passages were grown to confluence in RGEM medium (Renal epithelial cell growth medium, Lonza) in which a basal medium RBEM was supported with rhEGF (recombinant human EGF), transferrin, insulin, hydrocortisone, epinephrine, triidothyronine, and Fetal Bovine Serum (FBS), preserved at +4°C and changed every two days in the culture. Cells with medium were incubated at 37°C with CO_2_ at 5%. Once they became confluent, cells were exposed to C5a anaphylotoxin (10^−7^ M) at different times. After C5a stimulation, cells were washed 3 times in PBS1X and fresh medium was replaced. In addition, cells were exposed to H_2_O_2_ (100-300 μm) as positive control of senescence. For C5aR1 inhibition, mouse monoclonal anti-C5aR (Abcam) was pre-incubated for 1h before the C5a exposure. DNA and RNA were simultaneously extracted from RTEC using the AllPrep DNA/RNA Mini Kit (Qiagen) according to the manufacturer’s protocol. Nucleic acid concentration and quality were assessed using a NanoDrop Spectrophotometer (NanoDrop Technologies).

### DNA methylation analysis

DNA methylation analysis was performed utilizing the Illumina methylation profiling platform consisting of HiScanSQ system and Infinium HumanMethylation450 BeadChips. Bisulfite conversion of DNA extracted from RTEC was performed with the EZ-96 DNA Methylation Kit (ZymoResearch), and subsequent hybridization of this DNA was carried out on the Infinium Human Methylation 450 Bead Chips. Microarray data of the C5a stimulated RTEC and normal cultured RTEC are available under accession number GSE115227 at the Gene Expression Omnibus (GEO) (http://www.ncbi.nlm.nih.gov/geo/). All statistical analysis was performed using R and the RnBeads R package. Differential methylation analysis on site and region level was computed considering the difference in mean methylation levels C5a stimulated RTEC and normal cultured RTEC, the quotient in mean methylation and the t test assessing whether the methylation values in the two groups originate from distinct distributions. Additionally each site was assigned a rank based on each of these three criteria. A combined rank was computed as the maximum (i.e. worst) rank among the three ranks. Coverage of differential methylation analysis on region level was targeted across gene regions with sites in the promoter region, 5’-UTR, first exon, gene body and 3’-UTR in order to provide the broadest most comprehensive view of methylation state possible.

### Pyrosequencing assay

We validated the methylation status of differentially methylated CpG sites of CDK6, BAX, CYP1B1-AS1, BCL9, FZD6, CCDC6 genes using the pyrosequencing technology. The pyrosequencing assays (two PCR primers to amplify a specific target region and a sequencing primer) were designed with the Pyrosequencing Assay Design software (Biotage). The assays were set up sequencing control DNA samples chemically methylated and unmethylated respectively (EpiTect PCR Control DNA Set, Qiagen). The PCRs were performed using ImmolaseTM DNA Polymerase (Bioline, Aurogene) according to the manufacturer’s instructions. The amplicons of the specific targets were analyzed with a PSQ 96MA instrument (Qiagen). Sequencing reactions were performed with the Pyro Gold reagent kit PSQ 96MA (Qiagen) according to the manufacturer’s instructions and the sequencing analysis was conducted with the PSQTM 96MA software (version 2.02). The sequence of all primers, the PCR amplification conditions, and the sequence of the pyrosequenced fragments are indicated in Supplementary Table 3.

### Quantitative methylation-specific PCR assay (qMSP)

Bisulfite converted DNA was used to perform the qMSP by Methylamp MS-qPCR Fast Kit (Epigentek Group) according the manufacturer instructions. For each reaction 20 ng of bisulfite-treated DNA was used as template. Primer design and sequences and reaction methods are detailed in the Supplementary Methods section. Primers for the genes of interest were designed using MethPrimer (http://www.urogene.org/methprimer/index.html) (Supplementary Table 1). ACTB (β-actin) gene was used as a reference gene. No-template controls were included in each run as negative controls. An EpiTect Control DNA, a 100% methylated DNA (Qiagen), was used as a positive control for all genes studied. The PMR (percentage of methylated reference) (i.e. degree of methylation) was used to define the percentage of fully methylated molecules at a specific locus and was calculated as reported previously. Briefly, the PMR value was calculated by dividing the gene/ACTB ratio in a sample by the gene/ACTB ratio in SssI-treated leucocyte DNA (Qiagen) and multiplied by 100. Parallel PCRs were carried out for the genes of interest and reference. PMR values were detected using the comparative CT method. The relationship between the percentages of methylated DNA molecules and CT is described as PMR = 2−^DDCT^×100%.

### qPCR

qPCR was carried out with SsoAdvanced™ Universal SYBR® Green Supermix (Biorad) and the Light Cycler@96 (Roche). Cycling conditions and primer list sequence in Supplementary Table 4.

### SA-β-gal test

Senescence-associated SA-β-gal staining was performed as described in manufacturer's protocol (Cell Signaling Technology). After washing with PBS (pH 6.0), the cells were fixed in 4% paraformaldehyde for 15 minutes and stained with freshly prepared SA- β –gal solution (1 mg/mL X-gal, 40 mM citric acid/sodium phosphate (pH 6.0), 5 mM potassium ferrocyanide, 5 mM potassium ferricyanide, 150 mM NaCl, and 2 mM MgCl). Next, cells are incubated at 37 °C overnight in a dry incubator. The, we removed the staining solution, immersed the samples in 70% glycerol, and assessed the development of blue color. The number of SA-β-gal-positive cells was determined by counting at least 500 cells per dish (60mm) in five not overlapping fields. The % of the number of SA-β-gal strong positive cells that appeared with increased dimension is calculated as ratio considering the total numbers of cells counted in each dish.

### MTT assay

Primary epithelial tubular cells were incubated with and without human recombinant C5a for 3 or 24h followed by 24, 48 h of normal culture after medium culture change. Cultured RTEC proliferation was measured by MTT Cell Proliferation Assay Kit, according to the manufacturer instructions (Sigma Aldrich). Briefly, 3×10^4^cells/well were seeded in a 96-well plate, and then cells were treated with C5a as indicated. Absorbance at 570 nm was then measured by a spectrophotometer.

### Western blot

Protein lysates were homogenized by RIPA buffer with phosphatase and protease inhibitors. Proteins (30 μg) were separated in 4–15% polyacrylamide gel and then transferred to PVDF membrane (0.2mM) by Trans-Blot Turbo (BioRad, Hercules, CA). After blocking in BSA at 5%, the membranes were incubated overnight with the following primary antibodies: p21 (Abcam), p16INK4a (Abcam) and then with secondary antibody (hrp-conjugated, Santa Cruz). The same membrane was probed with mouse monoclonal anti-βactin antibody (1:20000; Sigma). The Electrochemiluminescence (ECL) system was used to detect the antibody binding, (Amersham, UK). The chemiluminescent signal was acquired by Chemidoc and quantified using Image J software.

### Statistical analysis

Graphs were displayed using GraphPad Prism Software 5. Data were expressed as median ± interquartile range (IQR) and compared with a Mann–Whitney test for tissue immunostainings. For qPCR, MTT and WB data were expressed as the mean± SD. Statistical analysis was assessed using unpaired Student’t-test. A p value of <0.05 was considered significant.

### Swine model of I/R injury

Animal studies were carried out under protocol approved by Ethical Committee of the Italian Ministry of Health**.** Briefly, I/R was induced by clamping the renal artery for 30min followed by reperfusion, as described previously. A biopsy was performed before ischemia (T0). Pigs were divided into two groups: control (CTRL, n=5, vehicle infused) and C1 Inhibitor treated group (C1-INH, n=5). Five minutes before the beginning of the reperfusion, rC1-INH was injected in the ear vein (500 U/kg). Biopsies were performed at 15, 30, 60 min and 24h after reperfusion. All animals were sacrificed 24 h after the procedure. Controlateral kidney was not removed.

### Immunohistochemistry

Renal sections underwent deparaffination and heat-mediated antigen retrieval (citrate buffer, pH=6.00) as previously described. For p16, p21 and βcatenin detection, sections were permeabilized with Triton 0.25% for 5 min, then blocked by Protein Block Solution (DakoCytomation, USA) for 10 min. Incubation was performed with antibodies against: p16, p21 (Abcam, Cambridge UK), IL-6 (Novus Biologicals), wnt4 and βcatenin. Then, the positive staining was detected by the Peroxidase/DAB Dako Real EnVision Detection System (Dako, Glostrup, Denmark). The peroxidase reaction was shown by a brown precipitate, counterstained with Mayers hematoxylin (blue). Negative controls were prepared by incubation with a control irrelevant antibody. Images were scanned by Aperio ScanScope CS2 device and signals were analyzed with the ImageScope V12.1.0.5029 (Aperio Technologies, Vista, CA).

### Cell cycle analysis

After treatment with C5a for 3h, 24h, 48h, RTEC cells were fixed in 70% ethanol then treated with 50 mg of RNase A/mL at 37 °C for 30 minutes. Aliquots of 300.000 cells were added to 0.2 mL of propidium iodide solution (from 50 μg/ml stock solution) with 0.1% sodium citrate and 0.1% Nonidet P-40) and incubated for 30 minutes in the dark at 4°C. The cellular DNA content was analyzed by flow cytometry FC500 flow cytometer (Beckmann Coulter) and analyzed by Kaluza software.

## Supplementary Material

Supplementary Figures

Supplementary Tables

Supplementary Table 1

Supplementary Table 2
