# Supplementary material for: Complement component C5a induces aberrant epigenetic modifications in renal tubular epithelial cells accelerating senescence by Wnt4/βcatenin signaling after ischemia/reperfusion injury
Source: Aging (Albany NY). 2019 Jul 8;11(13):4382–406. doi: 10.18632/aging.102059 (PMC6660044; doi:10.18632/aging.102059)
Supplement: Supplementary Figures [file aging-11-102059-s004.pdf]

SUPPLEMENTARY FIGURES

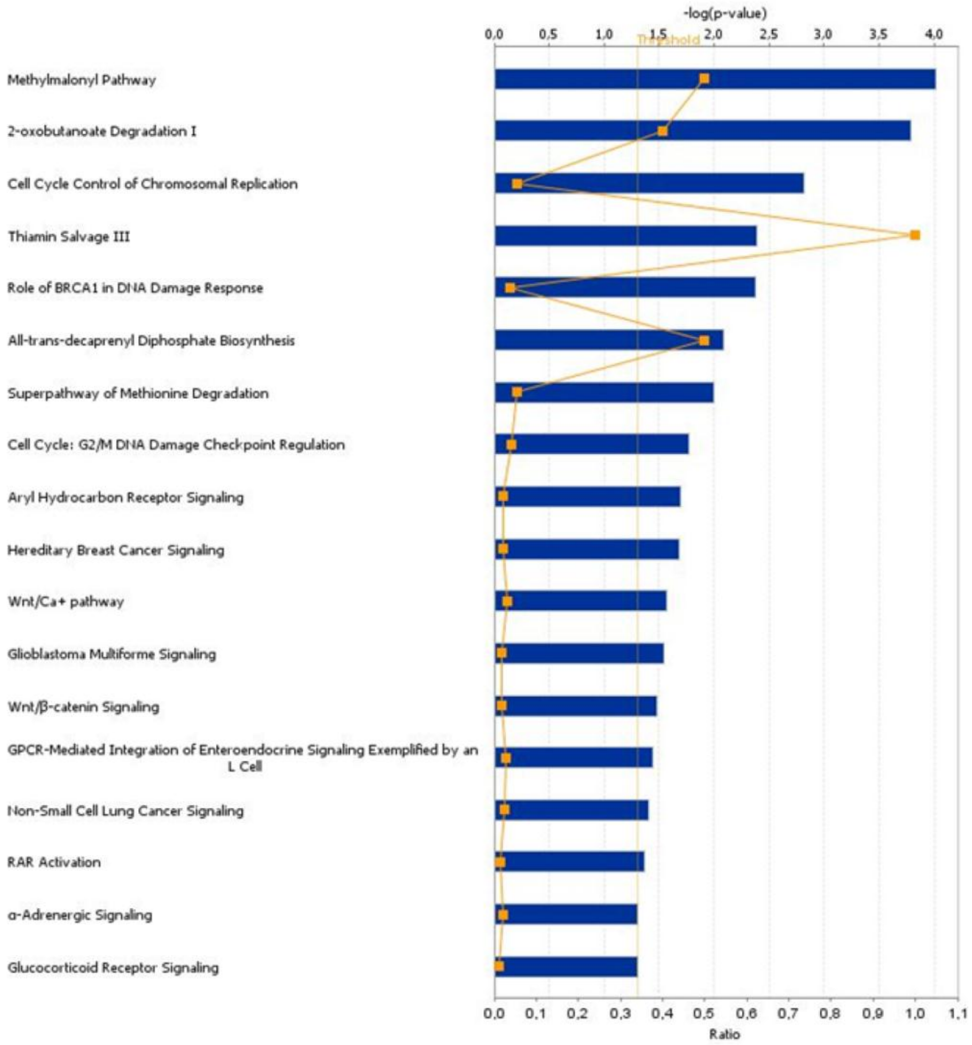

Supplementary Figure 1. Functional analysis of the biological processes significantly altered in regions hypo- and hyper methylated by C5a. Analysis of enriched biological functions by Ingenuity pathway analysis software on the basis of genes contained in differentially methylated regions. In the first 18 of the most significant pathway we detected cellular functions related to Cell Cycle control, Wnt/βcatenin pathway and Aryl Hydrocarbon Receptor Signaling.

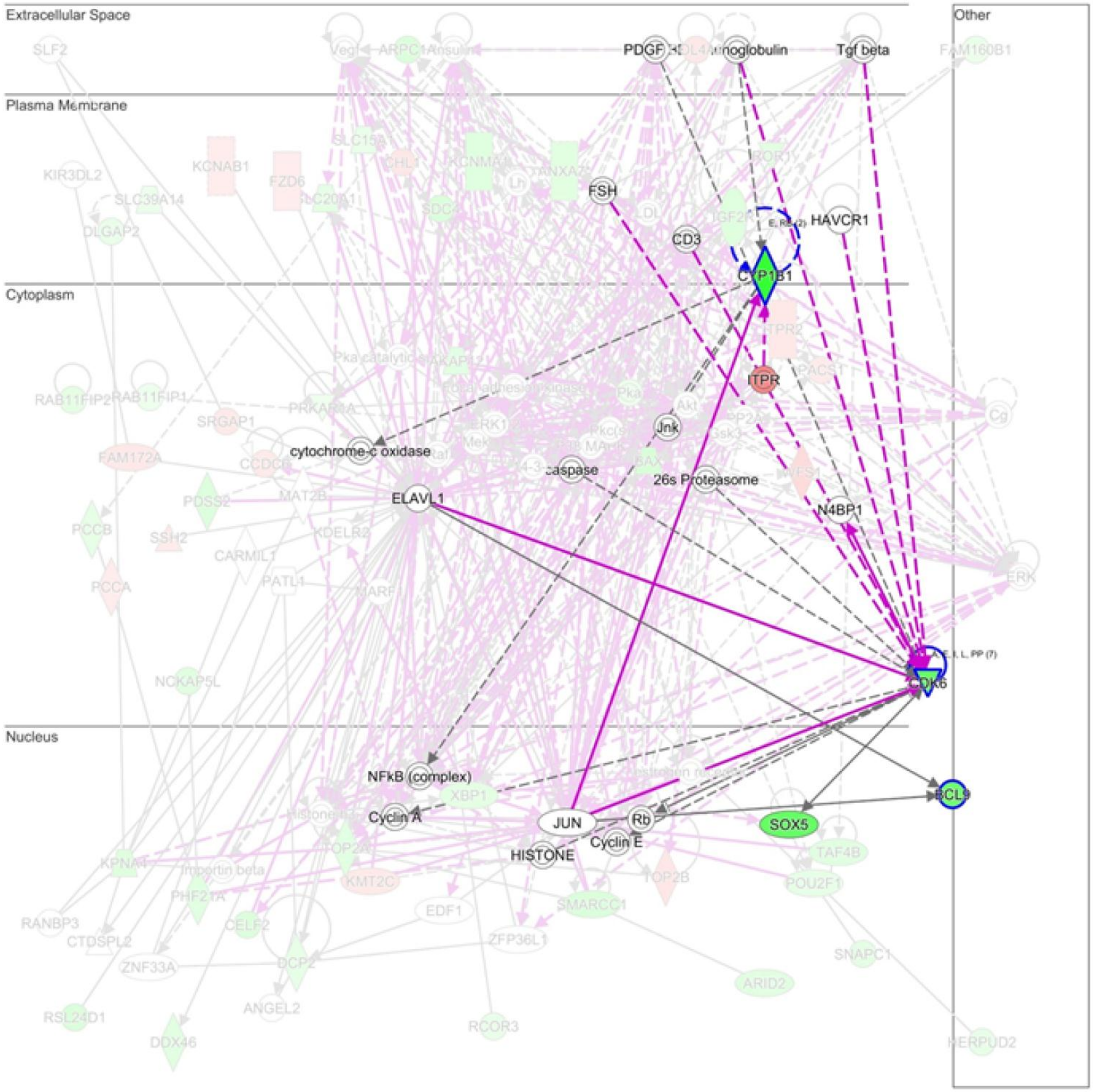

Supplementary Figure 2. Network analysis as determined on the basis of genes contained in differentially methylated regions. The network was algorithmically constructed by the ingenuity pathway analysis (IPA) software on the basis of the functional and biological connectivity of genes. The network is graphically represented as nodes (genes) and edges (the biological relationship between genes). Red and green shaded nodes represent up-methylated and down-methylated genes, respectively; others (empty nodes) are those that IPA automatically includes because they are biologically linked to our genes based on the evidence in the literature. Among network relationships, biological connectivity of CYP1B1, BCL9 and CDK6 has been highlighted as a central node.

Current Gene List: List_1
Current Background: Homo sapiens
93 DAVID IDs

Sublist	Category	Term	RT	Genes	Count	%	P-Value	Benjamin
☐	GOTERM_BP_DIRECT	resolution of meiotic recombination intermediates	RT		3	3,2	2,3E-3	7,3E-1
☐	GOTERM_BP_DIRECT	renal water homeostasis	RT		3	3,2	9,2E-3	9,2E-1
☐	GOTERM_BP_DIRECT	sister chromatid segregation	RT		2	2,2	1,4E-2	9,2E-1
☐	GOTERM_BP_DIRECT	mitotic DNA integrity checkpoint	RT		2	2,2	1,4E-2	9,2E-1
☐	GOTERM_BP_DIRECT	positive regulation of endoplasmic reticulum unfolded protein response	RT		2	2,2	2,2E-2	9,6E-1
☐	GOTERM_BP_DIRECT	short-chain fatty acid catabolic process	RT		2	2,2	2,2E-2	9,6E-1
☐	GOTERM_BP_DIRECT	DNA topological change	RT		2	2,2	4,0E-2	9,9E-1
☐	GOTERM_BP_DIRECT	response to salt stress	RT		2	2,2	4,0E-2	9,9E-1
☐	GOTERM_BP_DIRECT	snRNA transcription from RNA polymerase II promoter	RT		3	3,2	4,0E-2	9,8E-1
☐	GOTERM_BP_DIRECT	DNA unwinding involved in DNA replication	RT		2	2,2	4,4E-2	9,7E-1
☐	GOTERM_BP_DIRECT	positive regulation of apoptotic process	RT		5	5,4	4,7E-2	9,6E-1
☐	GOTERM_BP_DIRECT	mitotic recombination	RT		2	2,2	5,7E-2	9,7E-1
☐	GOTERM_BP_DIRECT	biotin metabolic process	RT		2	2,2	6,2E-2	9,7E-1
☐	GOTERM_BP_DIRECT	endoplasmic reticulum calcium ion homeostasis	RT		2	2,2	6,6E-2	9,7E-1
☐	GOTERM_BP_DIRECT	regulated exocytosis	RT		2	2,2	6,6E-2	9,7E-1
☐	GOTERM_BP_DIRECT	negative regulation of cell migration	RT		3	3,2	6,9E-2	9,6E-1
☐	GOTERM_BP_DIRECT	nucleosome disassembly	RT		2	2,2	7,4E-2	9,6E-1
☐	GOTERM_BP_DIRECT	neuron migration	RT		3	3,2	8,2E-2	9,7E-1
☐	GOTERM_BP_DIRECT	negative regulation of endoplasmic reticulum stress-induced intrinsic apoptotic signaling pathway	RT		2	2,2	8,3E-2	9,6E-1
☐	GOTERM_BP_DIRECT	protein targeting to Golgi	RT		2	2,2	8,7E-2	9,6E-1
☐	GOTERM_BP_DIRECT	spermatogenesis	RT		5	5,4	9,7E-2	9,6E-1

Supplementary Figure 3. Gene ontology analysis performed on the basis of genes contained in differentially methylated regions. The main biological functions are associated to DNA checkpoints, apoptosis regulation and chromatin state modifications.

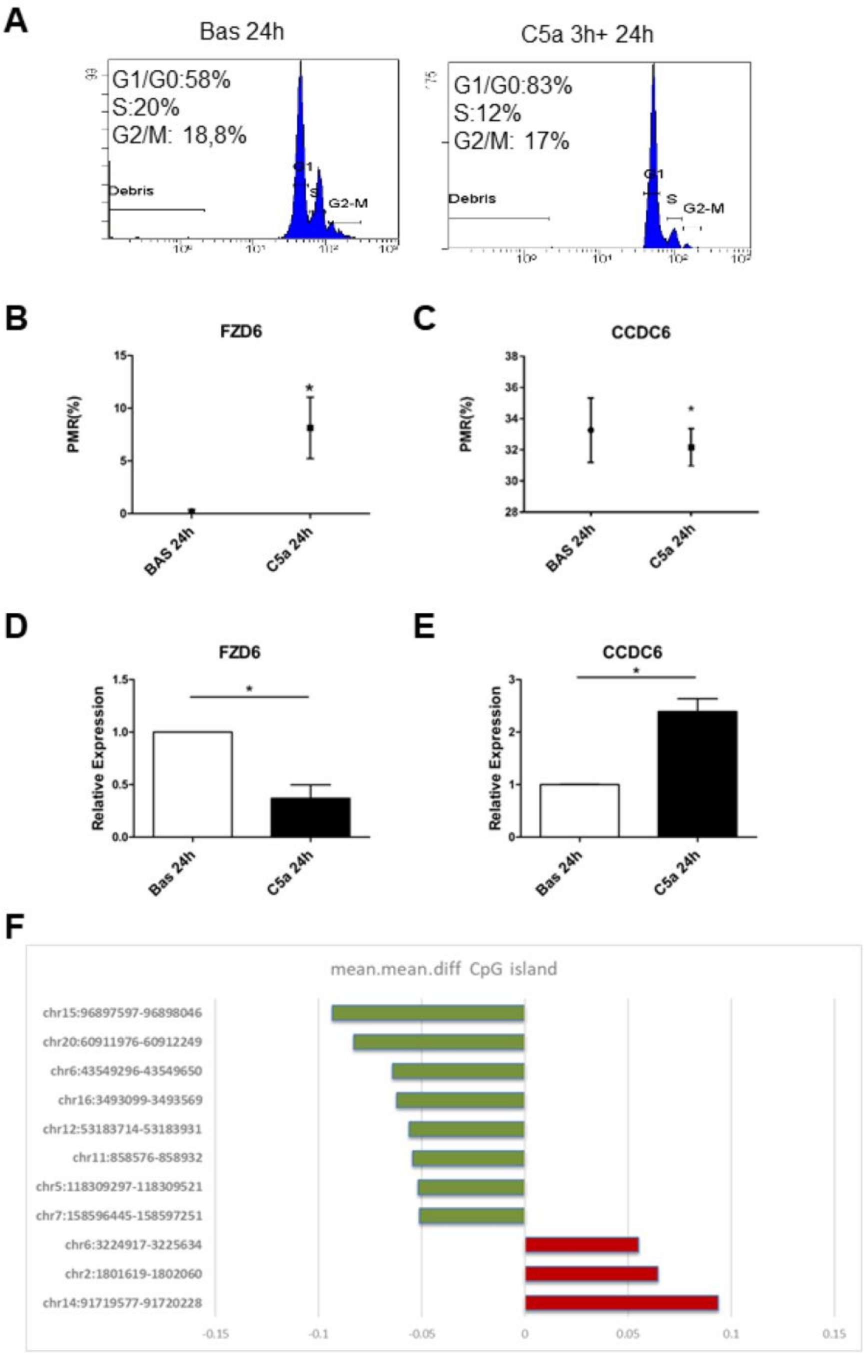

Supplementary Figure 4. Cell cycle analysis of RTEC treated with C5a after staining with propidium iodide (**A**, **B**). G0/G1, G2/M, and S indicate the cell phases and histograms indicated the DNA content distribution. (**B**) C5a stimulation lead to most cells halted in G0/G1 phase of the cell cycle (83% versus 58% of non-stimulated cells). (**C**) The DNA methylation status of FZD6 and CCDC6 DNA was determined by qMSP real-time analysis. The degree of fully methylated molecules at a specific locus was expressed as a PMR index. The percentage PMR was calculated as described in the Materials and methods. Qiagen methylation control DNA was used as full methylated reference. (**D**, **E**) Gene expression of FZD6 and CCDC6 evaluated by qRT-PCR in the C5a stimulated-RTEC and normal RTEC cultured for 24h. (**F**) Graph showing the CpG island differentially methylated between C5a-stimulated RTEC and unstimulated RTEC. CpG islands are annotated in the left. Mean values are represented as mean difference in mean values across all sites in a region. Hypermethylated CpG islands (n=3) are indicated in red, hypomethylated CpG islands (n=8) are indicated in green.
