# Supplementary material for: Complement component C5a induces aberrant epigenetic modifications in renal tubular epithelial cells accelerating senescence by Wnt4/βcatenin signaling after ischemia/reperfusion injury
Source: Aging (Albany NY). 2019 Jul 8;11(13):4382–406. doi: 10.18632/aging.102059 (PMC6660044; doi:10.18632/aging.102059)
Supplement: Supplementary Tables [file aging-11-102059-s003.pdf]

SUPPLEMENTARY TABLES

Please browse Full Text version to see the data of Supplementary Tables 1 and 2:

Supplementary Table 1: Tiling regions.

Supplementary Table 2: sites.

Supplementary Table 3. Primers and conditions for the pyrosequencing analysis.

	Pyrosequencing primers	Size (bp)	PCR annealing (°C)	Sequence to analyze
PYCYP1B1F	ATGGGGAYGGAGAAGGGTGTTT	100	55,7	
PYCYP1B1R	BIO-ACCCCCAATCTCAATCTCAAC			
PYCYP1B1S	GGAYGGAGAAGGGTG			TTTYGTTYGTYGAGTTTYGTTG TTTTTGG
PYBCL9F	gAAATAgAggATTTTTTTTTgAgA	233	54	
PYBCL9R	BIO-CCACAAAACATTTTCATATCACAT			
PYBCL9S	GAGGATTTTTTTTTGAGAA			YGTTTTTTAAATGTATAAAATA AAAT
PYCDK6(24)F	GATAAATTTTTGGAGTGTTTTTTG	193	50	
PYCDK6(24)R	BIO-CCAAAATAAAAAATTCATCATCAC			
PYCDK6(24)S	TTTTGAATTGTTAAGAGGA			ATAAYGTTATATAAAAATTTTT T

Abbreviations: F, forward; R, reverse; S, sequence; PY, pyrosequencing; BIO, biotinylated.
In red: CpG site of interest derived by Illumina platform.

Supplementary Table 4. Primers.

**qPCR**		
**Target**	**For 5'→3'**	**Rev 5'→3'**
CYP1B1	CCT CTT CAC CAG GTA TCC	ATG AGG AAT AGT GAC AGG C
BCL9	CCA TGA TGC TAT CAA GAC TGT G	CGA GGA TTC TGT GTA TTA ATG C
FDZ6	AAA GCA AAA GCT CGA CCA GA	GCC AGG CCA GTG TCA GTA AT
CCDC6	ACC ATC CAA GCC AGG GCT GA	CAA GAT GCT GTT CTA GTT CGG C
CDK6	TGC ACA GTG TCA CGA ACA GA	ACC TCG GAG AAG CTG AAA CA
MCP1		
CCN2 (CTGF)	TTGGCCCAGACCCAACTA	GCAGGAGGCGTTGTCATT

**MSP-primers**		
**Target**	**For 5'→3'**	**Rev 5'→3'**
CYP1B1	TCGGGAAGTAAGTTTAAGTCG	AAAAACGAAAATACCCACGTT
BCL9	TAGGATATAGGAGGCGGTTT	TAACCTAACTACGCACGCAAA
CDK6	AGGGATAAATATTAAGTCGTGAT	CTCCTCCTCCTTTACGAAAC
FZD6	GGGGGTCGTAATAGACGGTA	ACTCCGAAATTACGTCCGAA
CCDC6	GGAAGATGGGGTTATAATACGGT	AACCTCGCAAATACCGAAAA
